# The enigma of fine mobile structures on the aortic surface in a patient undergoing transcatheter aortic valve replacement: a case report

**DOI:** 10.1093/ehjcr/ytae263

**Published:** 2024-05-27

**Authors:** Gerald I Cohen, Karim Saleb, Patrick Troy, Klaus D Hagspiel, Thomas Lalonde

**Affiliations:** Department of Cardiology, Ascension St. John Hospital, 22101 Moross Road, Detroit, MI 48236, USA; IU Health Ball Memorial Physicians Cardiology, 2525 W University Ave. Suite 300, Muncie, IN 47303, USA; Department of Cardiology, Ascension St. John Hospital, 22101 Moross Road, Detroit, MI 48236, USA; Department of Radiology and Medical Imaging, University of Virginia Health System, 1215 Lee St. 1st Floor, Charlottesville, VA 22903, USA; Department of Cardiology, Ascension St. John Hospital, 22101 Moross Road, Detroit, MI 48236, USA

**Keywords:** Transoesophageal echocardiography, Aortic atherosclerosis, Ultrasound, Mobile aortic structures, 3D imaging

## Abstract

**Background:**

The surface of the aorta generally does not show motion unless mobile atheroma, thrombi, vegetations, or intimal flaps are present. We previously described unusual mobile filamentous structures in the carotid artery. Here, we describe similar findings in the aorta and their possible cause.

**Case summary:**

An 88-year-old female with progressive exertional dyspnoea and severe aortic stenosis had a successful transcatheter aortic valve replacement (TAVR). A filamentous structure was noted on the focused pre-operative 2D transoesophageal echocardiography in the proximal descending aorta and post-TAVR as long strand-like structures attached to the thickened intimal wall with a planar component on 3D imaging. These findings were not associated with symptoms or clinical sequelae on short- and long-term follow-up.

**Discussion:**

The mobile structures that we describe are atypical for atheroma, thrombi, vegetations, and dissections in terms of their form and clinical presentation. 2D imaging showed that the filaments had focal thickening and emerged from the aortic surface. These findings suggest a relationship with the intima, perhaps from atherogenesis or injury with disruption or lifting of the intimal surface. No clinical sequelae were detected that may also relate to their position in the descending aorta and not the arch.

**Learning points**

- 2D and 3D transoesophageal echocardiography can detect and define very small mobile elements on the surface of the aorta.
- These elements emerge from the intimal surface and may indicate intimal disruption or injury.
- This case report did not find clinical sequelae of these findings on long-term follow-up.

## Introduction

The surface of the aorta generally appears static on ultrasound except for gross mobile atherothrombosis, vegetations, and dissecting intimal flaps.^1–3^ We previously described unusual mobile filamentous structures at the common carotid artery–bulb junction in subjects with atherosclerosis or risk factors who were otherwise healthy.^4^ Here, we report the 2D and 3D findings of filamentous structures on the aortic intimal surface on transoesophageal echocardiography (TEE) in a patient undergoing transcatheter aortic valve replacement (TAVR) for severe aortic stenosis (AS) and the differential diagnosis of these findings.

## Summary figure

**Figure:**
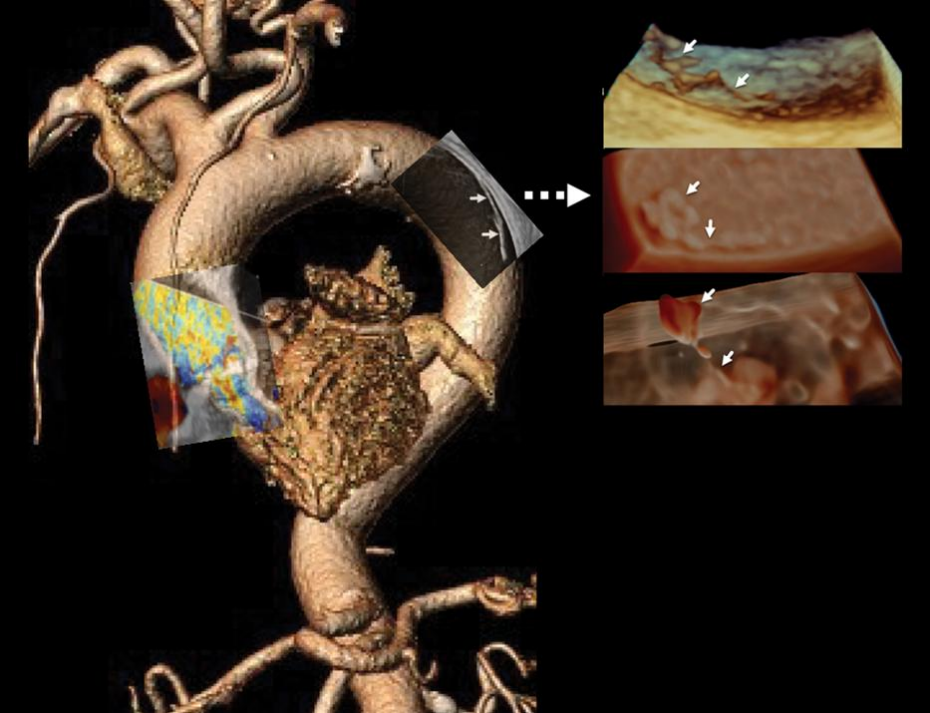


## Case presentation

An 87-year-old female presented with progressive dyspnoea while walking and a change in her chronic heart murmur. She had a history of hypertension, hyperlipidaemia, hypothyroidism, and falls. On physical examination, she was comfortable at rest and had a harsh 3/6 systolic murmur that radiated to her carotid arteries and an attenuated S2.

A transthoracic echocardiogram confirmed severe calcific AS with a Vmax of 4.3 m/s, a mean gradient of 44 mmHg, a valve area of 0.8 cm^2^, and mild regurgitation. Stroke volume index was normal at 49 mL/m^2^. Other findings included a hyperdynamic left ventricle with moderate hypertrophy, severe mitral annular calcification, moderate mitral regurgitation, and moderate increase in pulmonary artery systolic pressure. The patient was referred for TAVR. Seven weeks before the procedure, she had a coronary angiogram via the right radial artery that showed a 70% left anterior descending artery stenosis that was stented. Four weeks later, still chest CT images (GE Revolution CT 160 mm detector system, Milwaukee, WI) with contrast (Omnipaque 350) showed focal areas of aortic atheroma and no filamentous structures (Supplementary material online, *Video 1*).

**Figure 1:**
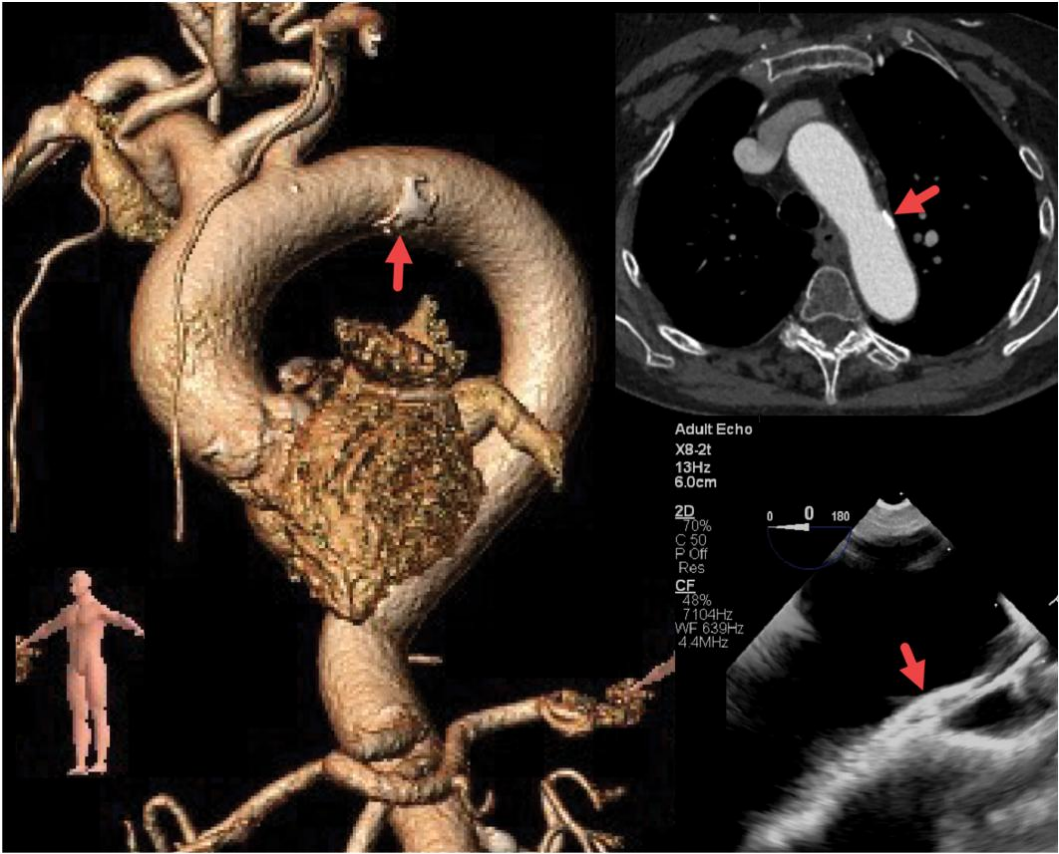
A focal atheroma was present in the distal arch on 3D rendering (left) and 2D (right upper) CT images. The aorta is unfolded. A <4 mm thick plaque was also visualized on a 2D long-axis TEE view of the arch (bottom right).

**Figure 2:**
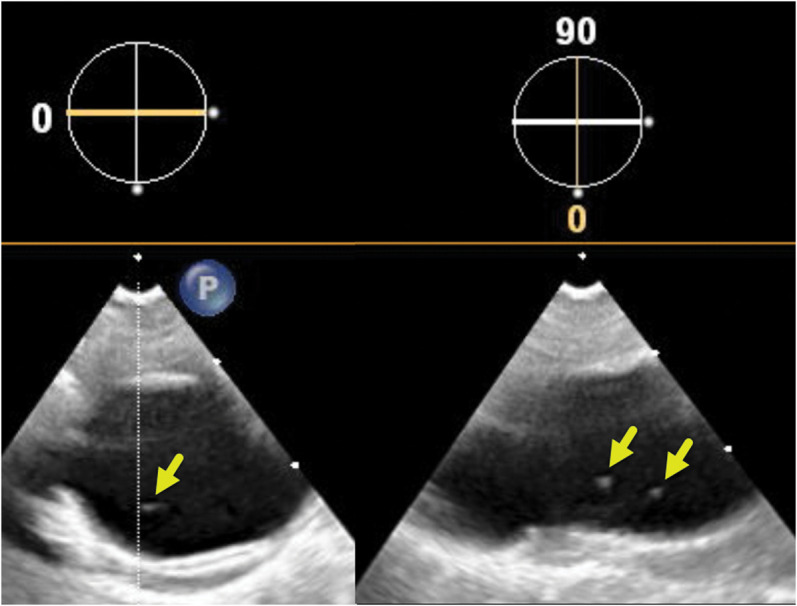
Fine mobile strand-like structures were faintly seen attached to the wall of the proximal descending aorta (arrows) on the short (left) and long axes (right) before TAVR implantation.

Three weeks after the CT scan, intraoperative TEE for TAVR (8 MHz, X8-2T probe, Philips Medical Systems, Bothell, WA) showed severe calcific AS with trivial regurgitation, moderate left ventricular hypertrophy, normal left ventricular ejection fraction, and left atrial enlargement. Moderate atheroma was noted in the aortic arch, which corresponded to the CT scan findings (*Figure 1*). No atheroma was detected elsewhere in the aorta. Limited aorta imaging showed fine mobile strand-like structures in the descending aorta (*Figure 2*, *Video 1*); a subtle finding that did not alter her management.

**Figure 3:**
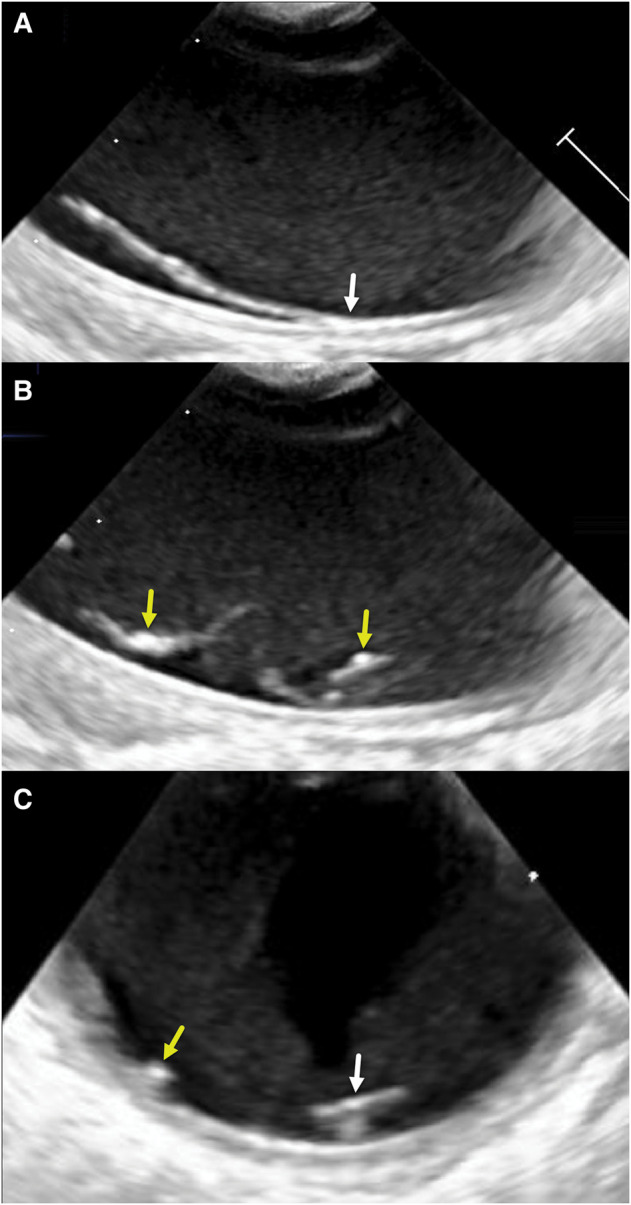
2D TEE images of fine mobile structures associated with the proximal descending aorta far wall. (*A*) Long-axis 2D views taken at 90° show a long strand in continuity with and emerging from the intimal surface (arrow). (*B*) Long-axis 2D view shows two strand-like structures with a single origin and areas of focal echogenicity arrows on the luminal side of the structures. (*C*) Short-axis 2D view taken at 0° also shows a focal area of increased echogenicity (left arrow) on the intimal surface and the planar nature (right arrow) of part of the structure (please also see the biplane image in *Video 2*).

**Figure 4:**
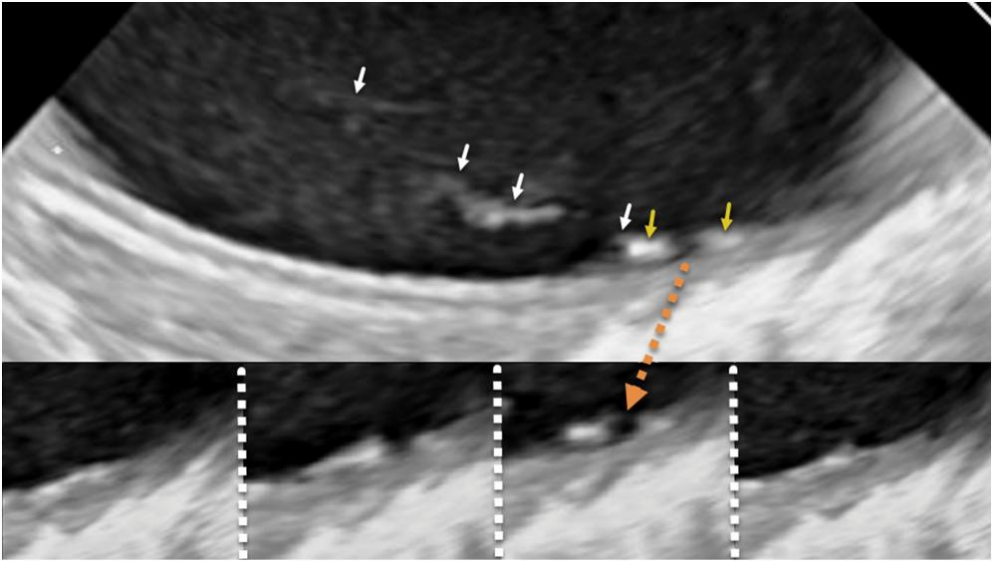
A filmstrip sequence of images illustrates a gap in the intimal surface that opens and closes. The two most rightward arrows point to intimal thickening that brackets the gap. The remaining four leftward arrows point to a strand-like structure that begins at a point of intimal thickening. This sequence is also demonstrated in *Video 2*.

The 26 mm EVOLUT PRO+ valve that was inserted showed normal function with a mean gradient of 8 mmHg, a valve area of 2.4 cm^2^, and no regurgitation (Supplementary material online, *Video 2*). Ventricular function was normal, and other findings were unchanged. The aorta was more extensively imaged post-TAVR with a focus on the mobile aortic abnormality that was noted at baseline on 2D TEE. Long, mobile, filamentous structures extended from one or more locations of the intimal surface (*Figure 3*, *Video 2*). Small focal areas of thickening were present on the luminal side of these structures, and, in one view, the intima showed surface mobility and interruption where the structures emerged (*Figure 4*, *Video 2*). The filamentous structures had a variable thickness and beaded appearance. Measurable diameters ranged from 0.2 to 1.3 mm but the thinnest areas were too thin or difficult to visualize for digital calipers to measure (Supplementary material online, *Figure 1*).

**Figure 5:**
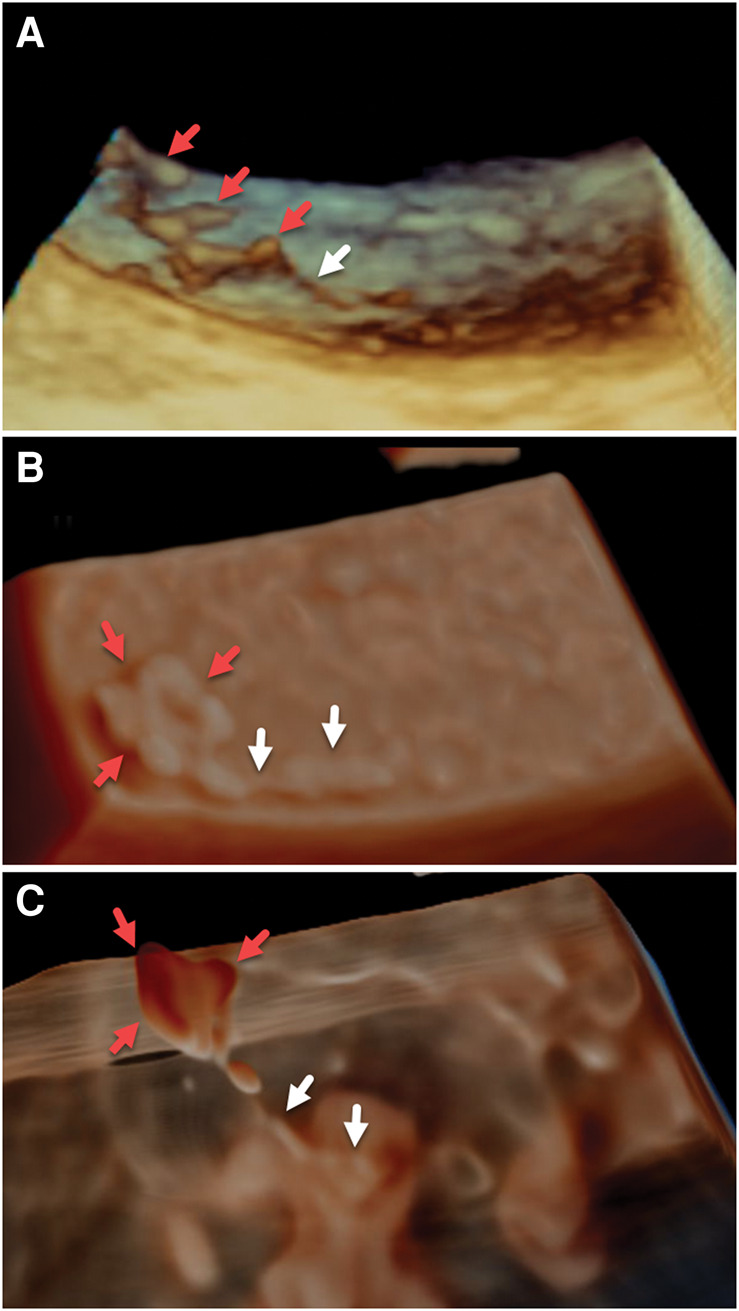
Long-axis 3D views of the surface of the descending aorta consist of standard (*A*) and 3D photorealistic and transillumination renderings (*B* and *C*). A network of fine mobile surface elements appears attached to the intimal surface with thinner strand-like [rightward arrow (A) or rightward two arrows (*B*, *C*)] and thicker nodular (*A*) or planar components (remaining leftward arrows) (*B* and *C*).

Photorealistic and transillumination 3D imaging demonstrated a network of filamentous structures and revealed a distal planar component at the end of one filament. The underlying intimal surface showed minor irregularity with multiple small mobile elements (*Figure 5*, *Video 3*).

These structures were not associated with symptoms or detectable clinical sequelae on short- and long-term follow-up. Her post-procedural course was punctuated by a fall and subdural haematoma at 6 months that was treated medically, and symptomatic complete heart block at 2 years that was treated with a permanent pacemaker. She recovered from these events and has been well and active, living at home independently.

## Discussion

We found that TEE can visualize mobile filamentous structures or tissue attached to the surface of the aorta. Several echo features indicated that these structures were related to the intima. Areas of focal thickening on the surface were consistent with minute atheroma, which would not be expected if the structures represented thrombi. In one view, the structure appeared rooted in the mobile surface where the intima was interrupted. Our findings are consistent with those of Belcaro *et al*. who coined the term ‘ultrasonic biopsy’ to indicate detailed examination of the intima media complex. He originally described early changes of carotid disease that included intimal surface interruption, focal intimal thickening, and media clouding.^5,6^

We previously described similar filamentous structures in the carotid artery in the setting of vascular screening with a 2D probe.^4^ These mobile filaments trailed from the common carotid artery–bulb junction distally and appeared as an extension of the intimal surface as if it was lifted. The subjects were healthy except for atherosclerosis or its risk factors. One subject developed sessile material in the bulb that disappeared on follow-up and initiation of statin therapy. This experience led us to look for filamentous structures in the aorta.

There were no clinical manifestations of mobile atheroma, thrombus, vegetation or dissection in our patient. Mobile aortic plaque, for example, are typically associated with complex underlying disease and may be large but can have linear features as described by Willens *et al*.^2^ in patients with blue-toe syndrome. In our structural heart practice, we observe mobile plaque mostly in elderly TAVR patients with severe atherosclerotic disease. The findings in this case report are different; the mobile filaments were finer than mobile atheroma and were located in the descending aorta where no significant plaque was present. Three-dimensional TEE also showed that the structures were part of a larger complex and more diffuse process, revealing planar features of the structures that were not readily evident on 2D imaging.

At first, our pre-TAVR images suggested that these structures were an incidental finding. Our subsequent unpublished experience indicates that these filaments may also form at the same location where TEE shows large guide catheters rubbing against the proximal descending aortic wall. Our patient’s aorta may have also been vulnerable to superficial injury because it was very unfolded that may have increased catheter contact with its surface during passage from the descending aorta to the arch. Atherosclerosis and amyloidosis are associated with severe AS and potential predisposing factors to intimal surface degeneration or injury.^7^

A small aortic dissection is another possibility that echo can image as a subtle intimal irregularity or discontinuity.^8^ Most reports on minimal aortic damage involve CT imaging and our images also show abnormalities that might appear linear on CT like the findings in acute trauma patients with very small dissections and thrombi.^9–12^ However, our TEE images show a diffuse process with multiple long and very short mobile filaments and no false lumen to indicate a dissection.

Published still images of minimal aortic injury indicate that the submillimetre resolution of CT might enable the detection of the filamentous structures.^9–12^ However, there are reasons why CT may not have been able to demonstrate them in our patient. The CT exam was not tailored to look for this unanticipated finding. Only still images were obtained; the subtle mobile findings may be difficult to appreciate without cine. Compared to TEE measurements, parts of the filaments would be below our CT scanner resolution of 0.23 mm. Visualization might also depend on a non-standard CT plane to visualize the continuity of the structure from where it attached to the aortic wall and extended into the lumen. We were not able to visualize the filaments after supplemental CT post-processing. Lastly, the filamentous structures may not have been present when the CT scan was performed weeks before the TAVR.

### Limitations

The mobile aortic structures appeared larger and more extensive after the TAVR than before, but we could not be certain that there was a change because the pre-procedural imaging was limited. In addition, there was no follow-up TEE to demonstrate the natural history of the finding. There was no pathological specimen to determine histologic correlation.

## Conclusion

Ultrasound can reveal the dynamic nature of the arterial surface, such as diffuse mobile filamentous structures on 2D and 3D TEE of the aortic wall. Their cause is difficult to determine but may be due to disruption of arterial surface integrity, atherogenesis, or injury, though no clinical sequelae were detected in our patient. Improvements in TEE resolution and multi-modality imaging and awareness of these findings may yield more information on their clinical associations, prevalence, and aetiology.

## Supplementary Material

ytae263_Supplementary_Data

## Data Availability

The data underlying this article are available in the article and in its online Supplementary material.

## Lead author biography

Dr Cohen graduated from McGill University and did additional training at the Ottawa Heart Institute in Canada. He has directed echo labs for 30 years and interventional echo at Ascension St. John Hospital for 11 years in Detroit and is Professor of Clinical Medicine at Wayne State University in Detroit. Special interests include atherosclerosis imaging, new IT methods for remote patient monitoring, and software design. He is a naturalist who loves the outdoors with his family and friends and his dog.
