# Supplementary material for: The enigma of fine mobile structures on the aortic surface in a patient undergoing transcatheter aortic valve replacement: a case report
Source: Eur Heart J Case Rep. 2024 May 27;8(6):ytae263. doi: 10.1093/ehjcr/ytae263 (PMC11210065; doi:10.1093/ehjcr/ytae263)
Supplement: ytae263_Supplementary_Data [file ytae263_Supplementary_Data.zip › Supplemental Material Legends.docx]

Supplemental Material Legends:

Video 1 - supplemental: Transverse, coronal and transverse CT images of the distal aortic arch and proximal descending aorta. The aortic wall shows focal areas of atheroma and minute irregularity but no evidence of filamentous structures.

Video 2-supplemental: Long (top) and short (bottom) 2D TEE images of the aortic valve before (left) and after (right) TAVR implantation.

Figure 1 - supplemental: Measurements of a filamentous structure that shows its variable thickness from 0.2 to 1.3 mm. The thinnest areas (arrow) were below measurement resolution and not well visualized.
