# Supplementary figures and images for: The enigma of fine mobile structures on the aortic surface in a patient undergoing transcatheter aortic valve replacement: a case report
Source: Eur Heart J Case Rep. 2024 May 27;8(6):ytae263. doi: 10.1093/ehjcr/ytae263 (PMC11210065; doi:10.1093/ehjcr/ytae263)

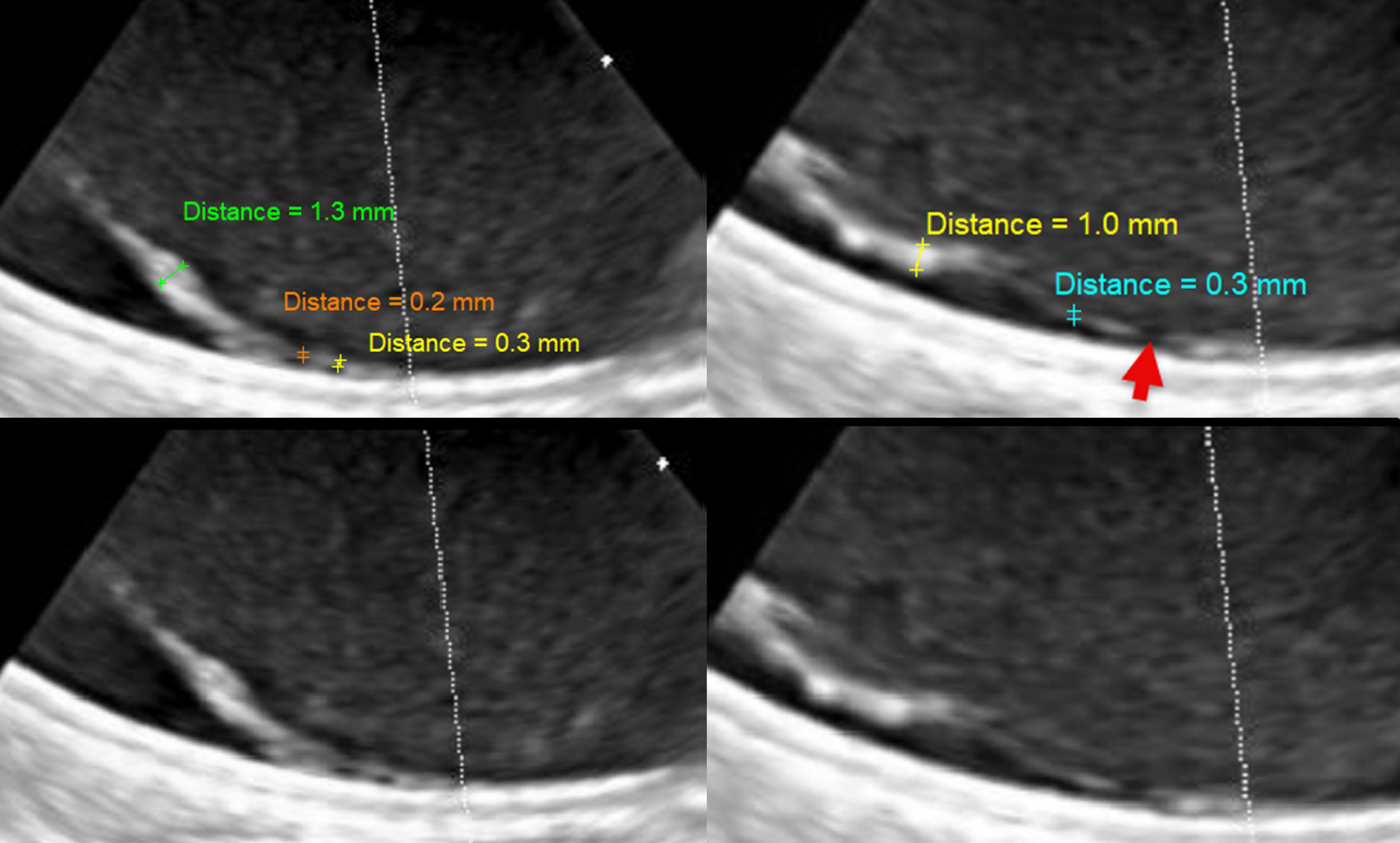

Supplement: ytae263_Supplementary_Data [file ytae263_Supplementary_Data.zip › Figure 1 (suppl).tif]
